# Cardiac dysfunction in Multisystem Inflammatory Syndrome in Children: An Italian single-center study

**DOI:** 10.1186/s13052-021-01189-z

**Published:** 2022-02-08

**Authors:** Savina Mannarino, Irene Raso, Massimo Garbin, Elena Ghidoni, Carla Corti, Sara Goletto, Luisa Nespoli, Sara Santacesaria, Elena Zoia, Anna Camporesi, Francesca Izzo, Dario Dilillo, Laura Fiori, Enza D’Auria, Annalisa De Silvestri, Alberto Dolci, Valeria Calcaterra, Gianvincenzo Zuccotti

**Affiliations:** 1 Pediatric Cardiology Unit, “V. Buzzi” Children’s Hospital, 20154 Milan, Italy; 2 Anesthesia and Intensive Care Unit, ``Vittore Buzzi” Children’s Hospital, 20154 Milan, Italy; 3 Pediatric Department, “V. Buzzi” Children’s Hospital, 20154 Milan, Italy; 4 Biometry & Clinical Epidemiology, Scientific Direction, Fondazione IRCCS Policlinico San Matteo, 27100 Pavia, Italy; 5 Department of Biomedical and Clinical Science “L. Sacco”, University of Milan, 20157 Milan, Italy; 6 Pediatric and Adolescent Unit, Clinical Pathology Laboratory , “Luigi Sacco” Hospital, 27100 Pavia, Italy; 7 Pediatric and Adolescent Unit, Department of Internal Medicine, University of Pavia, 27100 Pavia, Italy

**Keywords:** Multisystem inflammatory syndrome, Children, Heart, Cardiac, COVID-19

## Abstract

**Background:**

Multisystem inflammatory syndrome in children (MIS-C) is a novel condition temporally associated with SARS-CoV2 infection. Cardiovascular involvement is mainly evident as acute myocardial dysfunction in MIS-C. The aim of this study was to describe the cardiac dysfunction in patients with MIS-C, defining the role of severity in the clinical presentations and outcomes in a single cohort of pediatric patients.

**Methods:**

A single-center retrospective study on patients diagnosed with MIS-C, according to the Center for Disease Control and Prevention (CDC) definition, and referred to Vittore Buzzi Children’s Hospital in Milan from November 2020 to February 2021. Patients were managed according to a local approved protocol. According to the admission cardiac left ventricular ejection fraction (LVEF), the patients were divided into group A (LVEF < 45%) and group B (LVEF ≥45%). Pre-existing, clinical, and laboratory factors were assessed for evaluating outcomes at discharge.

**Results:**

Thirty-two patients were considered. Cardiac manifestations of MIS-C were reported in 26 patients (81%). Group A included 10 patients (9 M/1F, aged 13 years [IQR 5–15]), and group B included 22 patients (15 M/7 M, aged 9 years [IQR 7–13]). Significant differences were noted among clinical presentations (shock, diarrhea, intensive care unit admission), laboratory markers (leucocytes, neutrophils, and protein C-reactive), and cardiac markers (troponin T and N-terminal pro B-type Natriuretic Peptide) between the groups, with higher compromission in Group A. We found electrocardiogram anomalies in 14 patients (44%) and rhythm alterations in 3 patients (9%), without differences between groups. Mitral regurgitation and coronary involvement were more prevalent in group A. Total length of hospital stay and cardiac recovery time were not statistically different between groups. A recovery of cardiac functioning was reached in all patients.

**Conclusion:**

Despite significant differences in clinical presentations and need for intensive care, all of the MIS-C patients with significant cardiac involvement in this study completely recovered. This suggests that the heart is an involved organ and did not influence prognosis if properly treated and supported in the acute phase.

## Background

Multisystem inflammatory syndrome in children (MIS-C), also called pediatric inflammatory multisystem syndrome, is temporally related to SARS-CoV2 infection. It is a rare but severe hyperinflammatory disease that affects pediatric patients, typically 3–6 weeks after SARS-CoV2 contact [1].

While children are largely spared from severe acute SARS-CoV2 infection, probably due to under-expression of ACE2 receptors [2], MIS-C is a condition with clinical features similar to incomplete Kawasaki disease, macrophage activating syndrome, toxic shock syndrome, and septic shock [3, 4]. It affects multiple organs, the cardiac, gastrointestinal, muco-cutaneous, respiratory, neurological, and hematological systems [5, 6].

MIS-C was first described in April 2020 in Europe, but nowadays reports and papers exist from all over the world. The incidence is generally unknown, but a recent US paper reported an adjusted estimated incidence from 1 to 10 cases per 1.000.000 people per month [7]. Male patients and Hispanic and African people have a higher prevalence [8, 9].

The majority of patients are previously healthy children, without relevant comorbidities, with the exception of asthma and obesity [5].

The pathophysiological mechanism of the disease is unclear. MIS-C cases have a 3–6-week lag from the peak of cases in adults. The proposed mechanism is a post-infectious phenomenon mediated by IgG antibodies. Apparently, acute infection of SARS-CoV-2 induces a proinflammatory reaction and, in a genetically susceptible child, a delayed hyperinflammatory reaction of vasculitis with augmented levels of lymphocyte T-helper 17 and T-helper 1 mediators, and a cytokine storm, including massive release of inflammatory mediators and exaggerated activation of the immune system [10]. Chang et al., in a recent study, supported the role of a skewed cytokine profile towards IL6/IL8 pathways in causing heart dysfunction [11].

The clinical manifestations include persistent fever and involvement of at least two organ systems. Gastrointestinal symptoms, including vomiting, diarrhea, and abdominal pain, are reported in the vast majority of cases, from 50 to 100%. Rash, mucositis, conjunctivitis, and peripheral edema are frequently observed, in 36–81% of cases. Neurological symptoms are usually mild (headache, confusion, and irritability) and occur in 12–56% of cases, but rarely can be more severe, including meningitis and seizures. Respiratory symptoms are not a significant part of the MIS-C presentation, but shortness of breath, cough, hypoxia, and respiratory distress have been documented in up to 65% of cases [9, 12, 13].

Cardiac system involvement is frequently described and echocardiography, electrocardiography, and even laboratory abnormalities included. Acute myocardial dysfunction is the most prevalent, with generally mild or moderate depressed ejection fraction (EF). Only one third of patients present with severely reduced EF (EF < 30%). Cardiogenic or vasodilatory shock are described in up to a half of MIS-C diagnoses, and require inotropic support, while 28% need mechanical circulatory support [5].

Coronary arteries dilation and aneurysms are frequently described, but the prevalence varies significantly among reports, and it is not always easy to understand the rate of coronary enlargement and involvement. The most recent cardiological review reports an incidence of 8–24% in the larger studies. Most of the coronary involvement is mild (dilation or small aneurisms, Z score 2.5–5) and transient, but there have been a few reports of large aneurysms (Z score > 10) [14].

The aim of this study was to describe cardiac involvement in patients with MIS-C, defining the role of severe cardiac dysfunction in determining the clinical presentation and outcomes in a single cohort of pediatric patients.

## Patients and methods

### Patients

We carried out a retrospective study at the Pediatric Department of the Children’s Hospital, Vittore Buzzi, Milano, from the 1st of November 2020 to the end of February 2021. We included all children and adolescents who met the criteria of MIS-C diagnosis according to the Center for Disease Control and Prevention (CDC) [15].

For all patients, demographics and clinical characteristics, comorbidities, clinical presentation (fever duration, rash, conjunctivitis, extremities changes, mucositis, abdominal signs (diarrhea, vomiting, both), shortness of breath, headache, convulsions, shock), information about hospitalization (hospital stay length, need for pediatric intensive care unit (PICU), ventilation support, outcomes), and laboratory data (white blood cells, neutrophils, lymphocytes, C-reactive protein, N-Terminal pro B-type Natriuretic Peptide (NTproBNP), troponin T (high sensibility), ferritin, D-dimer, platelet, hemoglobin, interleukin-6) were recorded.

A cardiologic evaluation was carried out, including echocardiographic examinations and an electrocardiogram.

Mortality, length of hospital stay and LV systolic function at the discharge are considered as outcomes.

The study was conducted according to the guidelines of the Declaration of Helsinki, and approved by the Institutional Review Board of the Vittore Buzzi Hospital (Protocol number n. 2021/ST/004). All participants or their responsible guardians were asked for and gave their written consent after being informed about the nature of the study.

## Methods

### Clinical parameters

The patient’s physical examination included evaluation of weight, height, BMI (calculated as body weight in kilograms divided by height in meters squared) and pubertal stage according to Marshall and Tanner, as previously reported [16].

### Cardiologic assessment

For all patients, we considered echocardiographic ventricular measurements at admission before and after intravenous immunoglobulin (IVIG) therapy and during the hospitalization if necessary, considering cardiac electric abnormalities and arrhythmias including heart block, and at discharge.

Left ventricular (LV) function was qualitatively and quantitatively assessed by echocardiogram (Philips Affinity 70 or Vivid S5 GE Healthcare), and the left ventricular ejection fraction (LVEF) was calculated according to the Simpson’s biplane method [17].

To decide the therapeutic dosage of steroids, left cardiac function was classified as follows: normal (LVEF > 55%), mildly reduced (LVEF 45–54%), moderately reduced (LVEF 35–44%), or severely reduced (LVEF < 35%), according to our protocol. We also measured mitral regurgitation (qualitative and semi-quantitative evaluation), the degree of pericardial effusion (mild if ≤5 mm, moderate if > 5 mm), and the coronary artery size [18] (Montreal Z-score [19]).

According to the degree of compromised myocardial function, the patients were divided into two groups:

- Group A, patients with moderate and severe depression of the LVEF (LVEF < 45%);
- Group B, patient without signs of cardiac involvement or with only mild depression of the LVEF (LVEF ≥45%).

### Protocol therapy for MIS-C

In all patients, our standard protocol for treatment [18, 20], based on a review of the current literature [21] and mainly derived from Kawasaki disease experience and multidisciplinary expert consensus, was adopted. The rationale of the therapy was to control and stop the hyperimmune response of MIS-C.

The cardinal treatment consisted of IVIG at 2 g/kg. Corticosteroids (methylprednisolone) were added in case of hemodynamic impairment, need for oxygen, and heart failure. In the case of mildly reduced LVEF or minimal oxygen support we gave methylprednisolone 2 mg/kg for 5 days, followed by gradual tapering. If there was a significant oxygen requirement, mild organ injury, and/or moderately reduced LVEF, methylprednisolone 10 mg/kg for 1 day then 2 mg/kg for 5 days was given before tapering. If there was a need for respiratory support, inotropic support, moderate to severe organ damage, and/or severe hearing dysfunction, we used a high methylprednisolone dose of up to 30 mg/kg for 3 days.

Supportive care (inotropes, fluid resuscitation, diuretics, oxygen and ventilation, antibiotics, anticoagulation, or anti-thrombotic prophylaxis) were considered in most patients, accordingly to clinical multidisciplinary team evaluation. The anti-thrombotic prophylaxis was started in all patients > 12 years old, and was considered in patients < 12 years old, if the D-dimer was high (> 5 times the upper normal value) or if there was at least one known risk factors for thromboembolism. The anticoagulation therapy was prescribed in case of thrombosis and in case of severe LV dysfunction. With the reduction of the D-dimer or at the normalization of the LV function, heparin was shifted to low-dose aspirin for 3–4 weeks.

### Statistical analysis

Quantitative values are described as the mean and standard deviation (SD), or the median and interquartile range if not normally distributed (Shapiro–Wilk test). Qualitative variables were described as counts and percentages. Comparisons between groups were made with a chi-square test for qualitative variables, and with a t-test or Mann–Whitney test for quantitative data.

## Results

### General population data

We identified 32 consecutive patients. The median age at admission was 10 years, ranging from 5 to 15 years (interquartile range, IQR, 7–13). The features of the patients are reported in Table 1. Fever was present in all patients, with a median duration of 6 days at diagnosis. Nineteen patients (41%) presented with more than two organs involved. No major common preexisting comorbidities were recorded. Four patients (13%) were overweight and two patients (6%) were obese. None of the reported patients had congenital heart disease or preexisting cardiovascular disease. All patients had positive IgG serology for the virus.

**Table 1:** Baseline characteristics of the population, according to the degree of compromised myocardial function

Baseline characteristics	Total	Group A LVEF < 45%	Group B LVEF ≥45%	***p***-value
**Patient numbers (n)**	32	10	22	
**Age (years), median (IQR)**	10 (7–13)	13 (5–15)	9 (7–13)	ns
**Gender**				ns
**Male, n (%)**	24 (75)	9 (90)	15 (68)	
**Female, n (%)**	8 (25)	1 (10)	7 (32)	
**Overweight, n (%)**	4 (12.4)	2 (20)	2 (9.5)	ns
**Ethnicity, n (%)**				ns
Caucasian	24 (75)	6 (60)	18 (82)	
African	2 (6.3)	1 (10)	1 (5)	
Asian	1 (3.1)	0 (0)	1 (5)	
Hispanic/Latino	5 (15.6)	3 (30)	2 (9,5)	
**Organs ystems involved, n (%)**				ns
2	13 (40.6)	4 (40)	7 (32)	
3	10 (31.3)	3 (30)	8 (36)	
4	8 (25)	3 (30)	5 (23)	
5	1 (3.1)	0 (0)	2 (9)	
**RT-PCR positive, n (%)**	2 (6.3)	2 (20)	0 (0)	0.03
**Serology-positive, n (%)**	32 (100)	10 (100)	22 (100)	–
**Fever, n (%)**	32 (100)	10 (100)	22 (100)	–
**Fever duration (days)**	6	5.3	6.9	0.02
**Gastrointestinal n (%)**	30 (94)	10 (100)	20 (91)	ns
Vomit	19 (59)	5 (50)	14 (64)	ns
Diarrhea	20 (63)	9 (90)	11 (50)	0.04
Abdominalpain	19 (59)	6 (60)	13 (59)	ns
**Dermatological (rash), n (%)**	14 (44)	6 (60)	8 (36)	ns
**Shock, n (%)**	8 (25)	6 (60)	2 (9)	< 0.01
**Conjiuntivitis, n (%)**	18 (56)	5 (50)	13 (59)	ns
**Respiratory, n (%)**	13 (41)	5 (50)	7 (32)	ns
**Extremitychanges, n (%)**	7 (22)	3 (30)	4 (18)	ns
**Changes in the lips and oral cavity, n (%)**	8 (25)	3 (30)	5 (23)	ns
**Neurological, n (%)**	5 (16)	1 (10)	4 (18)	
Headache	3 (9)	1 (10)	2 (9)	ns
Convulsion	3 (9)	1 (10)	2 (9)	ns
**Hospital stay (days)**	13	14.5	11.5	ns
**Pediatric Intensive Care Unit admission, n (%)**	22 (69)	10 (100)	12 (54,5)	0.01
**Pediatric Intensive Care Unit stay (days)**	2	4.6	2	0.02
**Non invasive ventilation, n (%)**	12 (37.5)	5 (50)	7 (31.8)	ns
**Ventilation (days)**	2.5	3.2	2.9	ns
**Inotropic support, n (%)**	11^a^ (34.4)	7 (70)	4 (18.2)	< 0.001

*LVEF* Left ventricular ejection fraction; *PICU* Pediatric intensive care unit

^**a**^All of them were supported with epinephrine, and 2 received also norepinephrine and levosimendan

As expected, laboratory testing (Table 2) showed elevated inflammatory markers (C-reactive protein, fibrinogen, ferritin, D-dimer), neutrophilia with lymphopenia, and increased cardiac biomarkers.

**Table 2:** Biochemical characteristics of the enrolled patients, according to the degree of compromised myocardial function

Laboratory variable	All	Group A (***n*** = 10)	Group B (***n*** = 22)	p
White blood cells (n°/mm3)	9350 (6620–13,740)	13.740 (11.073–17.555)	7.130 (6.260–9.390)	0.001
Neutrophil (n°/mm3)	8050 (5240–11,770)	12.150 (9.470–14.630)	5.600 (4.875–8.235)	0.001
Lymphocytes (n°/mm3)	840 (650–1110)	1.005 (720–1.620)	750 (595–995)	ns
Platelets (n°/mm3)	165,000 (112750–219,500)	185.500 (163.000–230.000)	144.500 (102.000–191.000)	ns
Hemoglobin (g/dl)	10.8 (10.23–11.95)	10.6 (10–12)	11 (10–12)	ns
Ferritin (μg/L)	875 (389.25–1498.5)	788 (374–1.924)	1.007 (419–1.470)	ns
C-reactive protein (mg/L)	200.8 (119.48–279.3)	283 (261–308)	178 (92–241)	0.008
Troponin T (ng/L)	37 (14–74)	72 (40–243)	22 (8–49)	0.01
NT-proB-type Natriuretic Peptide (ng/L)	10,194.5 (1562–15,441.3)	14.825 (11.340–17.810)	5.921 (1.114–11.243)	0.01
D-Dimer (μg/L)	2777 (2126–5418)	2.855 (2.195–4.996)	2.756 (2.146–5019)	ns
Iinterleukin-6 (ng/L)	159 (97–405)	253 (156–579)	110 (96–155)	ns

All patients (100%) were treated with IVIG at the time of diagnosis, with a median delay of 5 ± 2 days from the onset of symptoms. Treatment with steroids (methylprednisolone) was added for 30 patients (93.8%): 5 (15.6%) received the highest dose, 10 (31.3%) the intermediate dose, and most (46.9%) the lower dose, according to our protocol.

Low-molecular-weight heparin was prescribed for 29 patients (91%), of whom 7 (22%) received a therapeutic anticoagulation dosage.

All patients were discharged with low dose aspirin and 93.8% with oral corticosteroid.

The majority of patients (69%) were admitted to the PICU. No patients required intubation and mechanical ventilation, while 37.5% needed non-invasive respiratory support.

### Cardiac involvement

Cardiac manifestations of MIS-C were reported in 26 patients (81%) and included ventricular dysfunction, valvular regurgitation, coronary artery dilation, arrhythmia, and conduction abnormalities.

The echocardiogram showed 9 (28%) children with preserved EF, 13 (41%) with mildly reduced function, and 5 (16%) and 5 (16%) with moderately and severely reduced function, respectively. Mitral regurgitation was present in 23 cases (72%), of whom 1 (3%) was severe and 4 (13%) moderate.

Only one child (3%) developed a transient coronary ectasia of the main left, with a Boston Z score < 2.5.

We found electrocardiogram anomalies in 14 patients (44%), without a difference between groups. ST segment changes and/or negative T waves were recorded in 12 (38%); 3 patients (9%) developed a type-1 Brugada pattern, one of which also presented with atrial fibrillation (AF) during placement of the central venous catheter, electrically cardioverted. One patient showed a type 2 BrS pattern. None of them had a positive family history neither for sudden cardiac death nor for Brugada syndrome.

Rhythm anomalies occurred in three patients (9%), including first-degree heart block, escape junctional rhythm alternating with sinus rhythm with premature ventricular contractions, and a short coupling interval. One patient presented with atrial fibrillation.

Ventricular dysfunction normalized in all our children in a median of 3 ± 2 days after treatment. Arrhythmias and electric abnormalities also tended to resolve.

### Cardiac involvement according to severity of dysfunction

In our population, 10 patients belonged to group A and 22 to group B. There were no significant differences in terms of baseline characteristics between the two groups, Table 1.

Group B had a less frequent presentation in shock at admission (group A 60% vs group B 9%, *p* < 0.01) and lower incidence of diarrhea (group A 90% vs group B 50%, *p* = 0.04). Group A presented with a shorter duration of fever (5.3 vs 6.9 days, *p* = 0.02).

All patients from group A were admitted to the PICU due to shock (100% vs 55%, *p* = 0.01) and/or significant cardiac dysfunction. Compared to group B patients admitted to the PICU, group A patients spent more time in the PICU (4.6 days vs 2 days, *p* = 0.02), and with a higher need for inotropic support (70% vs 18%, *p* < 0.01), mainly epinephrine. There were no differences between the groups in terms of mortality, ventilation support (*p* = ns), and hospital stay (*p* = ns).

In the pretreatment echocardiographic examination, group A showed a significantly increased prevalence of mitral regurgitation (90% vs 64%, *p* < 0.01); the prevalence of pericardial effusion was not different between the groups. In only one group A patient was a coronary dilation recorded; however, it was transient and did not reach the criteria for aneurysm. For further details, see Table 3.

**Table 3:** Echocardiographic findings pre treatment according to the degree of compromised myocardial function

Echocardiographic findings	Group A	Group B	***p***-value
LVEF≥55%, n (%)	0 (0)	9 (41)	< 0.001
LVEF 45–54%, n (%)	0 (0)	13 (59)	< 0.001
LVEF 36–44%, n (%)	5 (50)	0 (0)	< 0.001
LVEF ≤35%, n (%)	5 (50)	0 (0)	< 0.001
Coronary dilation, n (%)	1 (10)	0 (0)	–
Mitral regurgitation, n (%)	9 (90)	14 (64)	< 0.01
Pericardial effusion, n (%)	4 (40)	3 (14)	ns

*LVEF* Left Ventricular Ejection Fraction

As reported in Table 2, a statistically significant difference was detected in the white blood cell count (*p* = 0.001), number of neutrophils (*p* = 0.001), value of C-reactive protein (*p* < 0.01), troponin T (*p* = 0.001), and NTproBNP levels (*p* = 0.01), with higher values in group A compared to B. On the contrary, the two groups did not differ in lymphocytes (*p* = ns), platelets (*p* = ns), hemoglobin (*p* = ns), ferritin (*p* = ns), D-dimer (*p* = ns), and IL-6 (*p* = 0.08) values.

**Fig. 1:**
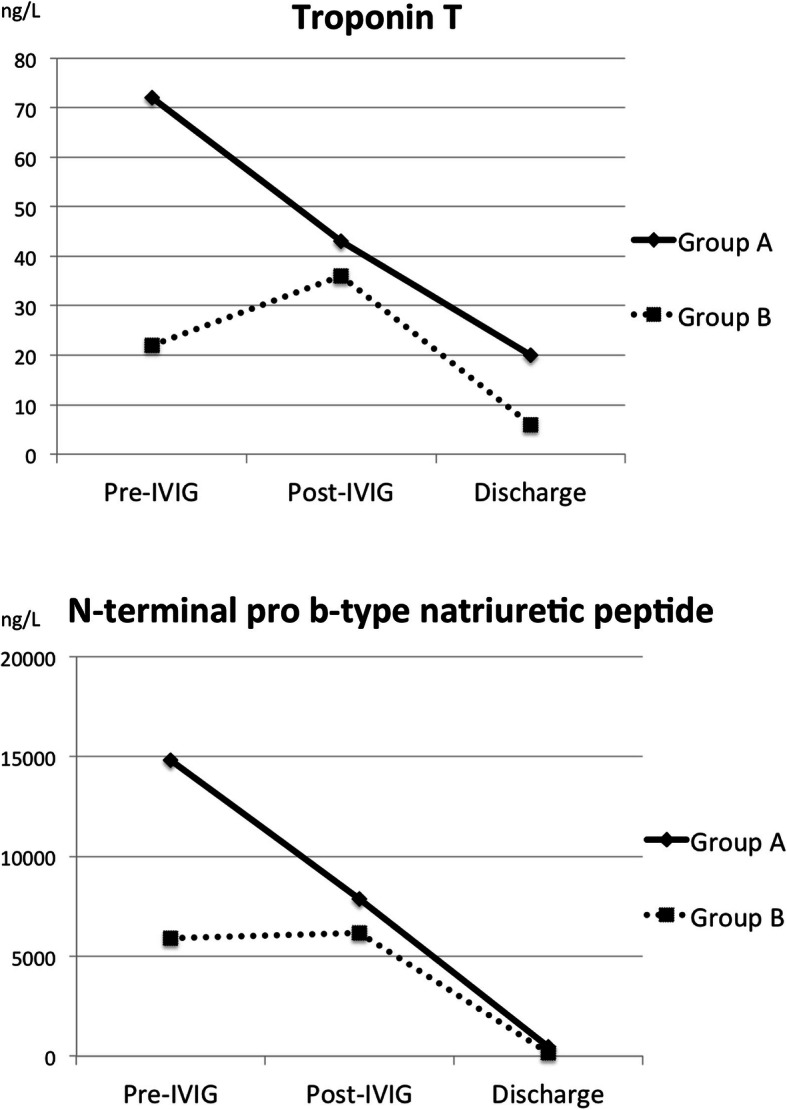
Trend of cardiac biomarkers in groups A (LVEF < 45%) and B (LVEF > 45%); during hospitalization . LVEF = left ventricular ejection fraction; IVIG = intravenous immunoglobulin

Pathological levels of troponin and NTproBNP were detected in all patients of group A. Furthermore, altered troponin and NTproBNP values were also noted in 33 and 77% of group B subjects, respectively. In Fig. 1, the trend of cardiac biomarkers in groups A and B during hospitalization were showed.

### Cardiac involvement during hospitalization

At the echocardiographic evaluation 24–48 h after the end the therapy with IVIG and during steroid therapy, all patients except one of group A (LVEF pre IVIG 33% after 35%) had an LVEF > 45%, without a significant mean difference between the two groups (group A 52.3% vs group B 55.4%, p = ns). Mild or moderate mitral regurgitation persisted in 60% (6/10) of group A and 64% (14/22) of group B, while mild pericardial effusion persisted in 2/10 (20%) patients of group A and in 4/22 (18%) of group B, p = ns.

At discharge, all patients had an LVEF > 55%, 10/32 (3%) a mild mitral regurgitation, and 6/32 (1.9%) a negligible pericardial effusion.

## Discussion

The recent pandemic of COVID-19 was characterized by poor clinical involvement of children compared to adults, but a novel MIS-C, mediated by cytokine activation, has been reported. This syndrome arises 3–5 weeks after no evidence of active current COVID-19 infection, and causes systemic multi-organ inflammation. In mid-May 2020, the CDC published a case definition for MIS-C for disease surveillance [15]. The heart is one of the main organs involved, and cardiovascular complications triggered recommendations for immunomodulatory treatments. Ventricular dysfunction, pericardial effusion, and valvulitis are commonly diagnosed in MIS-C.

We reported cardiac involvement in 81% of our patients, confirming that the heart, together with the gastrointestinal system, represents the most common target organ in patients with MIS-C. Only 28% of children showed a preserved EF, 41% patients had a mildly reduced EF, and 16 and 16% exhibited moderately and severely depressed functioning, respectively. A recent review of cardiac involvement in patients with MIS-C reported left ventricular dysfunction in 31 to 58% of patients [14]; similarly, in a recent study, Leora R. Feldstein et al. reported 34.2% of patients had a depressed LVEF [12].

The age distribution of our population supports a greater involvement of older children and adolescents, (median age of 10 years), as already reported in the literature [9, 22].

Fortunately, in accordance with other previous studies [23–25], we observed a prompt response to therapy (3 ± 2 days after treatment). At discharge, all patients had an LVEF > 55%.

It is known that most patients recover within days to weeks and mortality is rare, although the medium- and long-term sequelae, and particularly cardiovascular complications, are not yet known [14, 22, 26].

A systematic review and meta-analysis reported a rate of 28.1% of different types of ECG abnormalities as atrial or ventricular arrhythmias [27]. William Regan et al. showed ECG changes in a majority of children (67%) [28]. We found ECG abnormalities in 45% of patients, mainly abnormalities of repolarization, and one of the three cases with a type-1 Brugada pattern presented AF on the first day of hospitalization in intensive care. An Ajmaline test 8 months later confirmed Brugada Syndrome (not yet published). All patients at discharge presented a normal ECG, confirming a fast resolution of electrical anomalies and suggesting reversible damage.

In our pediatric cohort, only one Hispanic patient developed a transient ectasia of the main left coronary, which quickly resolved after therapy. There are conflicting descriptions about the data regarding the involvement of coronary arteries; the incidence of coronary artery dilatation/aneurysms varies significantly among reports (8–24%) [14, 29], and the pathological mechanism is, as yet, unknown. Abrams et al. showed some degree of association between muco-cutaneous alterations, conjunctival hyperemia, and coronary artery alterations, suggesting a possible overlap between MIS-C and Kawasaki disease [22]. None of our 23 patients with muco-cutaneous manifestations had coronary involvement; many factors could have contributed to this difference, such as a strict selection of patients (all showed serological positivity of IgG), a short interval between the onset of symptoms and therapy (median delay of 5 ± 2 days), combined therapy with steroids and intravenous immunoglobulin in almost patients (> 90%), and the prevalence of Caucasian ethnicity. Finally, the accurate recognition of patients with MIS-C may have limited overlap with Kawasaki disease.

We divided the patients in two groups according to severity of cardiac dysfunction on admission, to assess if there was any relationship between clinical and laboratory factors. All our patients had an alteration in the inflammatory index, with CRP, white blood cell, and neutrophil counts significantly higher in patients with more severe cardiovascular involvement; this suggests that myocardial involvement could be the hallmark of a hyperinflammatory state [5].

As expected, cardiac laboratory indices, including troponin T and NTproBNP levels, were higher in group A. Furthermore, values out of the ranges were also found in group B, suggesting that an underestimation of cardiac injury was not excluded using only echocardiographic evaluation.

Interestingly, the median value of NTproBNP was very high in group B as well. It is known that NTproBNP is associated with myocardial damage, stress, and fibrosis, and high NTproBNP levels constitute an independent diagnostic criterion for heart failure. However, it is also known that inflammation appears to be associated with natriuretic peptide release [30, 31]. Increasing NTproBNP in MIS-C without severe cardiac involvement may be related to the significant inflammatory state. Two patients in our series with shock at presentation showed a normal EF (52–60%) and troponin (49–46 ng/L), and had very high NTproBNP values (17.810–23.748 ng/L) and PCR (456–500 mg/L). As reported, shock and hemodynamic compromise in MIS-C can occur with preserved cardiac function.

We noted that patients with more compromised heart function spent more time in resuscitation, however there were no differences between the groups in terms of mortality, ventilation support, and hospital stay. Additionally, LVEF recovery in group A patients did not differ from that in group B, indicating that the cardiac impairment at admission did not affect the therapeutic response. Even though, according to our protocol, more severe patients were treated with high-dose corticosteroids, the limited number of patients did not allow us to estimate the real effect of the different dosages.

### Limitations of the study

We recognize that the limited sample size limits the strength of the analysis. Moreover, our results are similar to the current literature. Additionally, we were unable to perform global longitudinal strain analyses at the admission and check cardiac involvement with MRI during hospitalization, therefore an underestimation of cardiac injury should be considered. Finally, evaluation of long term heart consequences may be useful to define the real impact on children’s cardiac health and cardiac MRI are likely indicated in the patients’ follow-up.

## Conclusion

Despite significant differences in clinical presentation and the need for intensive care, MIS-C patients with significant cardiac involvement completely recovered from illness without differences in term of cardiac function and length of hospital stay. There is a strict relationship between inflammatory state and cardiac impairment. We confirmed that the heart is one of the main target organs; however, when properly treated and supported in the acute phase, the cardiac involvement does not appear to affect the prognosis.

## Data Availability

The datasets used and/or analysed during the current study are available from the corresponding author on reasonable request.

## Abbreviations

AF: Atrial fibrillation
CDC: Center for disease control and prevention
IQR: Interquartile range
IVIG: Intravenous immunoglobulin
LVEF: Left ventricular ejection fraction
MIS-C: Multisystem inflammatory syndrome in children
NTproBNP: N-terminal pro b-type natriuretic peptide
PICU: Pediatric intensive care unit
SD: Standard deviation
